# Improving the organisation of maternal health service delivery and optimising childbirth by increasing vaginal birth after caesarean section through enhanced women-centred care (OptiBIRTH trial): study protocol for a randomised controlled trial (ISRCTN10612254)

**DOI:** 10.1186/s13063-015-1061-y

**Published:** 2015-11-30

**Authors:** Mike Clarke, Gerard Savage, Valerie Smith, Deirdre Daly, Declan Devane, Mechthild M. Gross, Susanne Grylka-Baeschlin, Patricia Healy, Sandra Morano, Jane Nicoletti, Cecily Begley

**Affiliations:** Northern Ireland Network for Trials Methodology Research, Centre for Public Health, Institute of Clinical Sciences B, Queen’s University Belfast, Royal Hospitals, Grosvenor Road, Belfast, BT12 6BJ UK; Centre for Public Health, Queen’s University Belfast, Belfast, UK; School of Nursing and Midwifery, NUI Galway, Galway, Ireland; School of Nursing and Midwifery, Trinity College Dublin, Dublin, Ireland; Midwifery Research and Education Unit, Hannover Medical School, Hannover, Germany; Medical School and Midwifery School, Genoa University, Genoa, Italy

## Abstract

**Background:**

The proportion of pregnant women who have a caesarean section shows a wide variation across Europe, and concern exists that these proportions are increasing. Much of the increase in caesarean sections in recent years is due to a cascade effect in which a woman who has had one caesarean section is much more likely to have one again if she has another baby. In some places, it has become common practice for a woman who has had a caesarean section to have this procedure again as a matter of routine. The alternative, vaginal birth after caesarean (VBAC), which has been widely recommended, results in fewer undesired results or complications and is the preferred option for most women. However, VBAC rates in some countries are much lower than in other countries.

**Methods/Design:**

The OptiBIRTH trial uses a cluster randomised design to test a specially developed approach to try to improve the VBAC rate. It will attempt to increase VBAC rates from 25 % to 40 % through increased women-centred care and women’s involvement in their care. Sixteen hospitals in Germany, Ireland and Italy agreed to join the study, and each hospital was randomly allocated to be either an intervention or a control site.

**Discussion:**

If the OptiBIRTH intervention succeeds in increasing VBAC rates, its application across Europe might avoid the 160,000 unnecessary caesarean sections that occur every year at an extra direct annual cost of more than €150 million.

**Trial registration:**

Current Controlled Trials ISRCTN10612254, registered 3 April 2013.

## Background

Concern has been expressed globally at the rising caesarean section rate. For example, the World Health Organization (WHO) recently noted that ‘caesarean section rates higher than 10 % are not associated with reductions in maternal and new-born mortality rates’ [38]. In the United States in 2012, the caesarean section rate rose for the twelfth consecutive year to 32.8 %, a proportional increase of 56 % since 1996 [24]. In Europe, the caesarean section rate increased between 1999 and 2005 in all European Union member countries for which data were available [5]. In 2010, Italy had a caesarean section rate of 38 %, whereas the rate was 17 % in the Netherlands, indicating wide variations in maternity care practices within Europe [12, 23]. Some of the reasons that have been offered for the continuing increase in caesarean sections include medico-legal issues, the increasing use of electronic foetal heart rate monitoring, and reduced training in operative vaginal and vaginal breech births [1, 2, 19]. Maternal request for caesarean section, that is, a caesarean section in the absence of medically indicated reasons, is a further frequently cited reason for increasing caesarean section rates, ranging from 2.6 % to 26.8 % of all caesarean sections [20, 32]. The most common reason, however, is repeat caesarean section following previous caesarean section [27], and this is a significant factor contributing to the overall increased caesarean section rates. Repeat caesarean section accounts for more than one-third of all caesarean sections in the United States [7].

Vaginal birth after caesarean (VBAC) is a safe alternative to repeat caesarean section, is preferred by most women and is deemed to be a key way of reducing overall caesarean section rates (Cunningham 2010). However, VBAC rates, which rose steadily across the world in the early 1990s, have declined dramatically again. In 2006, for example, approximately 8 % of women in the US who had a previous caesarean section had a VBAC in their next pregnancy (Cunningham 2010). Other reports indicate VBAC rates of between 30 % and 56 % [4, 37]. In Europe, wide variations in VBAC rates exist between and within countries, reflecting variations in maternity care practices and maternity care provision. For example, VBAC rates in Germany, Ireland and Italy have been found to be significantly lower (29 to 36 %) than those in the Netherlands, Sweden and Finland (45 to 55 %) [11]. Combined with the high caesarean section rates in some countries, such as Italy, this means that many women in Europe are unlikely to have a vaginal birth unless they indicate that they would prefer that over a caesarean section [10].

A number of reasons are offered for the variations in VBAC rates. These include medico-legal factors and the inaccessibility of tertiary care centres, but variations in caesarean section rates are more likely to reflect national and individual clinician’s approaches to clinical decision-making [18]. Of the women who do choose and are supported to have a planned VBAC, VBAC success is considerably higher (70 % to 87 %) than the average rates (Cunningham 2010, [13]). This indicates that VBAC is a real and viable option for most women with previous caesarean section.

Increasing the caesarean section rates in conjunction with declining VBAC rates is a maternity care issue of particular concern. If caesarean section rates continue to rise at the rate of recent years, the projected overall caesarean section rate by 2020 will be 56 % [36]. Considering the increased adverse maternal and neonatal outcomes associated with caesarean section [9, 15–17, 21, 25, 28, 37], this trend and its potential effects on the health of women and babies into the future requires more attention. A dedicated and concerted effort by women and clinicians has the potential to halt increasing caesarean section rates [3], increase VBAC rates [33] and improve the overall maternal and neonatal health and wellbeing of women and their babies.

The OptiBIRTH trial, conducted across different European health settings, involves unique collaborative efforts of women and clinicians. It seeks to enhance women-centred maternity care and, through the development and testing of an innovative complex intervention aimed at increasing VBAC rates in high caesarean section and low VBAC countries, reduce the fragmentation and lack of coherence in health service delivery. The OptiBIRTH trial will provide evidence to inform the organisation and provision of maternity care for many hundreds of thousands of women with previous caesarean section in Europe. Two systematic reviews of existing evidence have been published as part of the wider OptiBIRTH project: one on women-centred interventions and one on clinician-centred interventions for increasing VABC rates [22, 30]. These provide justification for this trial and assisted in the design of the OptiBIRTH intervention.

This protocol sets out the design of the OptiBIRTH trial and has been prepared in accordance with the SPIRIT guidelines for the protocol for a randomised trial [6]. Findings of the trial will be reported in accordance with the CONSORT recommendations [26].

### Aim

The aim of the OptiBIRTH project, of which this trial is one component, is to improve maternal health service delivery, and optimise childbirth by increasing vaginal birth after caesarean section through enhanced women-centred maternity care across Europe.

### Objectives

The objective of this trial is to compare the effects of an intervention that has been developed with the intention of maximising VBAC versus usual care. This will be done through a cluster randomised trial in maternity units in three European countries with relatively low VBAC rates. The intervention was developed within the OptiBIRTH project and includes an effort to develop communities of practice in the intervention sites through face-to-face and online activities.

### Registration

The OptiBIRTH trial was prospectively registered in the ISRCTN Registry before randomisation was done (ISRCTN applied for: 17 March 2013; ISRCTN assigned: 3 April 2013; and randomisation conducted: 7 April 2013). The trial was assigned the following number: ISRCTN10612254.

### Study setting

A total of 16 sites (maternity units) agreed to take part in the OptiBIRTH trial initially: five from Ireland, five from Italy and six from Germany. In order to be eligible for participation, each site had to have a VBAC rate of less than 35 % and to provide a letter from the lead obstetrician (and, when available, the lead midwife) stating that they were willing for their institution to join the study. However, one of the German sites was withdrawn from the trial because of failure to recruit any participants in the first 6 months.

## Methods/Design

### Design

This is a cluster randomised trial of maternity units in three countries with relatively low VBAC rates (Germany, Ireland and Italy). The units represent a variety of healthcare settings (small, medium and large units, with annual births of 2,000 to 8,500) in both urban and rural locations [14]. For the purposes of the randomisation, two maternity units in the same city in Germany were considered as a single cluster for the purposes of the trial.

### Pilot study

A pilot study was conducted from January 2014 in Germany and February 2014 in Ireland and Italy, with sites having been pre-randomised to the intervention or control (see below). This pilot was used to identify problems with the research design and trial processes, to refine data collection and to examine selection and enrolment processes. The pilot concluded in March 2014 in Ireland and in April 2014 in Germany and Italy. The main analysis for OptiBIRTH will exclude women recruited during the pilot trial. It will include data on women who gave their consent on or after 1 April 2014 in Ireland and 1 May 2015 in Germany and Italy.

### Randomisation

The unit of randomisation in the trial is the maternity unit (the ‘site’). Randomisation had to be done in advance of recruiting women to participate in the trial to allow appropriate preparations to be made in each site. Further, given the temporal nature of pregnancy, it is not possible to hold randomisation until all women in each participating site have been recruited, because some women would have given birth by the time the last woman would have been recruited. However, as with cluster trials in which the participants cannot be blinded to the intervention, once the sites had been randomised, it is possible that recruitment of women to the trial was influenced by the knowledge of whether or not they would receive the OptiBIRTH intervention at that site. Hence, the primary analyses for the trial (see below) will consider data at the level of each site as a whole, rather than at the level of the women who were approached and formally recruited.

Within each of the three countries, the maternity units were matched by their annual number of births and then by VBAC rate. They were matched in either pairs or triplets and then randomised 1:1 or 2:1 to intervention or control, respectively. The randomisation was done using the random number generator in Microsoft Excel [RAND], with the first site in each matched pair or triplet being assigned a random number between 0 and 1. If the number was below 0.500, the site was allocated ‘OptiBIRTH intervention’. If it was above 0.500, the site was allocated ‘control’. If the number had been exactly 0.500 the randomisation would have been done again, but this did not happen. Where a matched pair had been formed, the second site was automatically allocated the opposite of the first site. Where a matched triplet had been formed, if the first site was allocated to the control group, the other two sites were automatically allocated to the intervention group. If the first site was allocated to the intervention group, the randomisation was repeated for the second site, and the third site was automatically allocated to the opposite of the allocation for the second site.

The randomisations were done in the presence of a witness who was unaware of which site was associated with which code within the pairs and triplets until after the allocations were complete. Although we recognise that it is not possible to mask the allocation of the sites from people who are familiar with them, including pregnant women and practitioners, further information on the sites is not included in this published protocol, to reduce the possibility that this knowledge might influence the referral of pregnant women to the participating sites during the recruitment phase for the trial or the follow-up period for the primary outcome analysis (see below).

### Eligibility criteria for participants

To be eligible for inclusion, pregnant women at a participating site must meet the following criteria:

1. Be ≥18 years of age at the time of booking
2. Have had one previous caesarean section using a lower segment transverse incision (not a classical/high vertical incision)
3. Speak and understand a language for at least one of the trial countries (English, German or Italian)
4. Provide informed consent to participate in the study

If a woman is known to have a multiple pregnancy at the time of the booking, she is not eligible.

### Training and support for trial sites

Staff members at all study sites are proficient in maternity care provision and in caring for women with one previous caesarean section. They are provided with information on the trial by members of the research team, including the national postdoctoral researchers and principal investigators.

### Interventions

Participating sites were randomised to either intervention or control.

#### OptiBIRTH intervention

Sites randomised to the intervention group will receive a complex innovative programme of evidence-based antenatal strategies, incentives and activities to increase the empowerment, engagement and involvement of women with a history of one previous caesarean section. Full details of the OptiBIRTH intervention will be published in a separate paper but, in summary, it seeks to empower women and to develop a community of practice in the site, with the intention of increasing the VBAC rate. The package includes face-to-face educational sessions about VBAC for clinicians in each site, specially designed antenatal classes for the recruited women and an optional interactive website and applications, which will assist them in setting personal goals to achieve their optimal birth outcome. Building a community of practice at the site, which would be more favourable to VBAC, should impact all women at the site, not just those who attend the antenatal classes or access the online resources.

#### Control

Sites randomised to the control group will receive the current standard of care in that maternity unit.

### Process evaluation

The OptiBIRTH research team has developed a process evaluation plan to explore and document the implementation of the OptiBIRTH intervention, and how it was received by key stakeholders and to identify factors that could explain variation in outcomes across intervention sites. This evaluation will include details of the attendance of clinicians at their face-to-face educational sessions, the attendance of women at the antenatal classes and their use of the online resources. It will also include the gathering of information from both clinicians and women on their experiences with the OptiBIRTH intervention and the various activities and resources made available to them. In addition, the sites in which the intervention was implemented will be described, and findings will be used to assist in interpretation of the trial results. Such process evaluations are particularly important in cluster randomised trials, where a ‘standardised’ intervention may, in fact, be implemented in different ways or be the subject of different reactions [31]. A resulting ‘lack of effect’ may thus merely indicate problems with implementation rather than true ineffectiveness [8].

### Outcome measures

#### Primary

The primary outcome measure will be the VBAC rates for women with one previous caesarean section for each site comparing the years before and after the delivery of the intervention. This is calculated as the number of women who gave birth by spontaneous vaginal delivery or with the use of ventouse or forceps after a single previous caesarean section, divided by the total number of women who gave birth at the site after a single previous caesarean section.

#### Secondary

Secondary maternal outcomes collected at and shortly after birth are described below:

1. Use of health care and other resources
2. Labour onset (spontaneous, induced, etcetera)
3. Spontaneous rupture of membranes
4. Acceleration of labour (artificial rupture of membranes, oxytocin use)
5. Analgesia during labour
6. Length of labour
7. Mode of birth
8. Perineal trauma (1st, 2nd, 3rd, 4th degree tear; episiotomy)
9. Maternal morbidity (for example: post-partum haemorrhage, uterine rupture, wound breakdown, admission to intensive care unit, etcetera)
10. Maternal death
11. Length of postnatal hospital stay (days)

Secondary neonatal outcomes collected during pregnancy and at, and shortly after, birth are described below:

1. Fetal demise during pregnancy (miscarriage, stillbirth, intrauterine death)
2. Gestation at birth
3. Apgar scores at 1 and 5 minutes after birth
4. Umbilical arterial and venous cord pH and base excess
5. Neonatal resuscitation
6. Admission to neonatal intensive care unit
7. Neonatal mortality
8. Neonatal morbidity (for example: seizures, hypoxic-ischemic encephalopathy, intracranial haemorrhage, meconium aspiration syndrome, renal failure, etcetera)
9. Length of neonatal postnatal stay (days)

Secondary maternal outcomes collected at 3 months post-partum are described below:

1. Satisfaction (with the intervention, mode of birth and participation in trial)
2. Breastfeeding (initiation and over the first 3 months post-partum)
3. Quality of life (measured by SF-36)
4. Use of health care and other resources

### Sample size assumptions and estimates

The sample size for this cluster randomised trial was derived by adjusting an estimate for the sample size for an individually randomised trial to allow for clustering. This was done by inflating the estimate by the design effect given by 1 + (ñ-1)ρ, where ñ is the average cluster size and ρ is the estimated intra-class correlation coefficient (ICC) for this study. With a background proportion of successful VBAC of 25 % and an ICC of 0.05, 12 trial units would be required, each containing 120 participating women (840 women in the intervention group and 840 women in the control group), to detect a 15 percentage point difference in successful VBACs (that is, an increase from 25 % in the control group to 40 % in the intervention group), with power of at least 80 % and an alpha level of 0.05. If the true ICC values are less than 0.05, the power of the study will increase. To allow for a loss to follow-up of 20 % of women and the possibility that one site per country will drop out of the trial, 15 trial units (16 maternity sites) were randomised across three countries.

### Recruitment and consent

In order to maintain the integrity of the cluster randomisation, each woman in each site will be screened for eligibility for OptiBIRTH, using a pre-designed Trial Screening and Register Form. Although all those who are eligible could be considered to have been randomised, it will only be possible to use data for those who give their consent.

Following screening, women who are judged eligible for OptiBIRTH will be informed of the study verbally and will receive a ‘study information pack’, including a detailed information leaflet and a consent form. This will be done by the midwives who are providing their antenatal care and takes place at the earliest opportunity in the woman’s interaction with the maternity unit. If a woman presents too late in her pregnancy to avail herself of the antenatal classes or the online resources, she will not be approached about OptiBIRTH. Women, having had time to consider the study information and agreeing to participate in the trial, will sign and return the consent form to the local research office. On receipt of the signed consent form, women will be contacted by the local postdoctoral researcher (or designated hospital midwife) and provided with further details on accessing the trial processes (intervention sites only).

When seeking their consent, women will be offered the opportunity to participate in the trial at one of two levels, which relate to both their use of the various elements in the intervention and the provision of data. If the OptiBIRTH intervention succeeds in building a community of practice at the site which is more favourable to VBAC, this should impact all women at the site, not just those who choose the ‘full participation’ option and attend the antenatal classes or access the online resources.

The two levels of participation are as follows:

1. Full participation: women in the intervention sites would choose this option if they wanted to be able to attend the OptiBIRTH antenatal classes and access the online resources. In both the intervention and the control sites, women who choose this option are agreeing to complete the health surveys and a diary of healthcare expenses and to allow the OptiBIRTH researcher to access their healthcare records and those of their babies.
2. Routine data only: women choose this option if they are willing to give permission for the OptiBIRTH researcher to access the healthcare records for themselves and their baby, but do not wish to attend the antenatal classes or access the online resources (in the intervention sites) or to complete the health surveys and diary of healthcare expenses (in both intervention and control sites).

### Data collection

Pre-designed data extraction forms will be used to collect all study outcome data. These include forms for participant self-report of antenatal and postnatal health and wellbeing and healthcare resource use, as well as expenditure surveys and clinician-report labour and birth outcome data collection forms. Where relevant, the forms will be provided to the participants and they will be asked to complete and return them to their hospital. Other data (such as routine data on birth events) will be collected at each participating site by each country’s national postdoctoral researcher or a designated research assistant. Each national coordinating team will use specially created Microsoft Access forms to enter and store their data before submission of the data in encrypted form to the OptiBIRTH trial and data management centre. The relevant data are to be submitted for each woman at each stage in the OptiBIRTH trial (from screening through to the data gathered on the postnatal follow-up forms). Each country’s national coordinating/postdoctoral researcher will audit approximately 5 % of the records for each participating site during their visits to those sites.

### Data management and validation

Data will be submitted by encrypted email to OptiBIRTH’s trial and data management centre (Centre for Public Health, Queen’s University Belfast) monthly and stored in the trial database (Microsoft Access 2007). Appropriate validation rules are in place for each field in the OptiBIRTH data. Cross-validation routines have been established, where the content of one field would determine the validity of data in another field. For example, if a woman has a spontaneous vaginal birth, the fields for capturing data following a caesarean section should not be completed. The OptiBIRTH trial database will contain identifiable information and, as such, conforms to legal requirements as defined in the UK Data Protection Act 1998. In response to this Act, Queen’s University Belfast issued a Policy Statement and developed robust data protection recommendations that the Centre for Public Health at Queen’s University Belfast adhere to. Given that the database contains identifiable data, the potential risk associated with this database relates to a potential breach of participant confidentiality, whereby a party not involved directly in the study gains access to identifiable data, and the aforementioned policies and practices are designed to prevent this.

### Data analysis

Statistical analysis plans will be prepared for the analyses of both the clinical and the cost-effectiveness data, and made available separately to this protocol. Where possible, the main analysis will be by ‘intention-to treat’ but this is not always possible in a cluster randomised trial where, by the nature of the intervention, the recruiters and the potential participants are aware of their allocated intervention before they are asked to provide their consent. Therefore, the principal analysis to test the effect of the intervention at the site level will compare differences in the primary outcome, namely the proportions of VBAC, between the intervention and control sites. The comparison of VBAC rates in each of the participating sites will compare the change in the VBAC rates in the intervention sites versus the change in the control sites between the calendar year 2012 (that is, before the OptiBIRTH intervention was available to any hospital) and, if possible, the 12-month period after the month that the last OptiBIRTH baby is born in each hospital. However, for some hospitals, it might be necessary to use the data for the calendar year (that is, January to December) that follow the birth of the last OptiBIRTH baby in that hospital because of the practical difficulties of accessing hospital level data for periods other than calendar years.

In addition to the primary analyses at the level of the hospital, we will analyse the data for the women who agree to take part in the trial and their babies, in order to assess the effects of the intervention on the secondary outcomes listed above. Where outcomes have been collected as continuous variables (for example, duration of labour) they will be analysed as continuous data to compare the intervention and control sites. Likewise, dichotomous data (for example, breast feeding at 3 months) will be used to compare the proportions in the intervention and control sites. The analyses will adjust for whether the woman had had any prior vaginal birth or prior VBAC, and her BMI and age. We will conduct subgroup analyses for these adjustment variables and the three countries. Our hypothesis is that the effects of the intervention will not be so heterogeneous across these subgroups to invalidate the calculation of an overall result for the trial as a whole.

### Trial oversight

#### Trial management group

There will be monthly Skype or teleconferences of the Trial Management Group, comprising the principal investigators and postdoctoral researchers from each country, in addition to the principal investigator and data manager for the trial, the project coordinator for the OptiBIRTH project as a whole, and other members of the OptiBIRTH project as appropriate to the specific meeting.

#### Trial steering committee

The Trial Steering Committee (TSC) comprises the members of the overall Steering Committee for the OptiBIRTH project as a whole. This group meets quarterly, usually by Skype or teleconference but with one face-to-face meeting per year. Summary reports for each site, each country and the trial as a whole will be prepared every 3 months for the OptiBIRTH TSC, showing the data for each 3-month period and for the full duration of the trial to date. These reports contain data relating to the management of the trial to show its progress without revealing interim results.

#### Data monitoring committee

To optimise participant safety and the scientific integrity and credibility of the results of the trial, an international Data Monitoring Committee (DMC) will conduct an interim analysis of data from approximately 400 to 700 women who have birthed and will provide an interim report. They will be provided with unblinded data on the VBAC rate at each site in the year before the OptiBIRTH trial began, the number of women recruited in each site and the proportion of those who are known to have birthed who did so by spontaneous vaginal delivery or with the use of ventouse or forceps. This initial assessment of the effects of the intervention on VBAC and safety will influence the decision on whether control sites should be offered the intervention on completion of the trial within the available funding timeframe. The DMC will assess participant safety and whether either intervention is showing a much stronger or weaker effect than expected. It will make recommendations concerning the future of the study to the TSC.

### Ethical considerations

The ethical issues involved in this study are the key principles of ensuring the protection of human rights, the maintenance of scientific integrity, the minimising of harm and maximising of benefit and ensuring justice and equity, autonomy and informed consent, confidentiality, data protection and privacy. Ethical approval was granted by the Faculty of Health Sciences, Trinity College Dublin, Ireland and regional Research Ethics Committees for all participating study sites in each participating country. A list of the relevant committees was provided to the journal as part of the approval process for this manuscript, but, as noted above, identifying information for the sites is not included in this published protocol to reduce the possibility that this knowledge might influence referral of pregnant women to the participating sites during the recruitment or follow-up phases for the trial.

### Ancillary studies

Should the opportunity arise to conduct a SWAT (Study within a Trial) in OptiBIRTH, this will be discussed with the TSC and implemented with their approval. The SWAT programme has been developed by the Northern Ireland Network for Trials Methodology Research as a means to encourage the conduct of research within research, to reduce uncertainties about the most appropriate and effective methods to use in randomised trials and other evaluations of health and social care [34, 35].

### Dissemination

The main findings of OptiBIRTH will be published under an Open Access model and will be presented at relevant national and international conferences. The current intention is that the analyses for the participating women will be published following collection of the final outcome data for all of those women.

## Discussion

Recruitment to the OptiBIRTH trial is expected to close in all sites in 2015, with all participating women expected to birth by the end of the year. Recruitment has been slower than anticipated, in part because birth rates have fallen at several of the sites. As with cluster randomised trials in general, it has not been possible to recruit all potentially eligible women at each site. Among the reasons for this are that some of the women who were offered participation in the trial did not wish to join and because of challenges in fully initiating the study in each site. This may introduce differences between the participants in the intervention and control sites, which reinforces the importance of the primary analyses being a comparison of the VBAC rates for all women with one previous caesarean section at each site before and after the study period using routine data from each site. If the OptiBIRTH intervention succeeds in increasing VBAC rates, its application across Europe might avoid 160,000 unnecessary caesarean sections every year, which occur at an extra direct annual cost of more than €150 million.

## Trial status

This protocol was submitted for publication in May 2015 when recruitment was ongoing. The last women are expected to join the trial by October 2015.

## Abbreviations

DMC: Data Monitoring Committee
ICC: intra-class correlation coefficient
ISRCTN: International Standard Randomised Controlled Trial Number
SWAT: study within a trial
TSC: Trial Steering Committee
VBAC: vaginal birth after caesarean section
WHO: World Health Organization
